# Europe After COVID-19: A New Role for German Leadership?

**DOI:** 10.1007/s10272-021-0955-z

**Published:** 2021-04-06

**Authors:** Francesco Saraceno

**Affiliations:** Research Centre in Economics, Political Science, OFCE Sciences-Po, 69, quai d’Orsay, 75007 Paris, France

## Abstract

The German change of attitude concerning fiscal policy and the mutualisation of efforts to fight the pandemic is driven by self-interest, and as such might be structural.

A famous quote by Jean Monnet (1978) states that Europe will be "built through crises" and will be "the sum of their solutions" (417). Nevertheless, many agree that in recent years, the sovereign debt crisis has been "wasted" by European policymakers who have remained largely impervious to the discussion on the respective role of state and market in ensuring economic growth and convergence. Instead of learning from the "rethinking macroeconomics" debate triggered by the global financial crisis to build a less dysfunctional and more cohesive Europe, they locked the single currency in a vicious circle of deflationary policies and sluggish growth. Then came the reaction to the coronavirus pandemic, which was surprising both in size and in timeliness. It is as if the mistakes of previous years, somewhat metabolised, had prompted governments and European institutions to move without hesitation. This article traces the policy response to the COVID-19 crisis, highlighting the role of Germany in this change of perspective. It then investigates the reasons behind the end of the German "virtue" and analyses the implications for the current debate on eurozone reform.

## Building a dam against the COVID-19 tsunami

In March 2020, EU governments were the first line in the fight against the pandemic. This was inevitable: the EU is a union of sovereign states and neither public health nor fiscal policies are among its competencies. For the latter, consistent with the no taxation without representation motto, spending and tax decisions can only be made at the national level, where accountability with the voters lies. Measures to support households and businesses fell into three broad categories: firstly, support for health systems under stress (among other things, because of the systematic underfunding of the past decades); secondly, measures to preserve employment and support workers' incomes; and finally, measures to support the liquidity of companies, with tax deferrals and credit guarantees. Nearly everywhere, the measures were extended well into 2021 as the economic effects of the pandemic unfolded. The impact on public finances was immediate: In 2020, eurozone government deficit and debt increased to 8.4% and 98.1% of GDP respectively (IMF, 2021). The measures were fruitful, as everywhere income and employment have fallen significantly less than GDP. Germany was particularly proactive, with two large stimulus packages (in March and in June) that expanded the duration and scope of both short-time work schemes (*kurzarbeit*) and unemployment benefits, and provided loans and guarantees for liquidity strapped firms. The June package was aimed at boosting domestic demand through a temporary VAT decrease and incentives for investment in green technologies and digitalisation.

While member states were in the front line, European authorities promptly moved to protect them from market pressure. The Commission activated the Stability and Growth Pact (SGP) suspension clause and softened state aid rules, thus allowing states to pump money into the economy and to support the sectors hit by the pandemic. Meanwhile, the ECB launched an extensive pandemic emergency purchase programme (PEPP), later extended in size and in duration (until spring 2022). Together with the mass of savings available worldwide, this has kept interest rates low, contributing to debt sustainability. Finally, European institutions made loans available to member states for their urgent expenditures. Whether through an existing mechanism (the European Stability Mechanism (ESM) for health-related expenditure), or a newly created mechanism (the temporary Support to mitigate Unemployment Risks in an Emergency (SURE) for labour markets), the principle was the same: Europe borrows at favourable rates and transfers the loans to countries that can thus save on interest expenditure. SURE was extremely successful and in autumn 2020 started lending €90 billion to 18 countries. On the contrary, no country applied for ESM lending: Despite a relaxation of access conditions (the "pandemic line"), it remains an instrument aimed at ensuring the stability of the euro area in the event of financial crises; as such, it allows the European institutions to interfere in member states' budget processes. No country judged the limited gain in interest that the pandemic line would warrant to be worth this risk of interference.

© The Author(s) 2021. Open Access: This article is distributed under the terms of the Creative Commons Attribution 4.0 International License (https://creativecommons.org/licenses/by/4.0/).

Open Access funding provided by ZBW – Leibniz Information Centre for Economics.

**Francesco Saraceno**, OFCE – Sciences Po, Paris, France; and Luiss, Rome, Italy.

## The medium-term challenges: A proactive EU, but not (yet) a Hamiltonian moment

As we slowly emerge from the crisis, the EU is going from a mere supporting role to being a key player. The transition towards a sustainable growth path, the revamping of public investment, the rethinking of our welfare systems – these are challenges that not even the largest European countries can hope to meet efficiently on their own. Economies of scale and externalities militate in favour of policies implemented, or at least financed and coordinated, at the EU level.

The need to provide these European public goods is what inspired the Next Generation EU programme, which combines the Recovery and Resilience Facility (RRF) and other mechanisms with the European multiannual budget, endowing member states with a total of €1,850 billion over seven years. The innovative aspects of the instrument have been thoroughly discussed. First, the issuance of common debt for significant amounts (€750 billion) to finance an extensive investment programme aimed at channelling the recovery within the EU's long-term objectives (green growth, digitalisation, social cohesion); then, the allocation of resources to member states based on the needs that have emerged from the pandemic rather than from the usual allocation keys (which is why Italy, usually a net contributor to the EU budget, will be a net beneficiary of the RRF). The debt will be repaid from 2028 to 2058, hopefully thanks to an increase of own resources (the web tax, the carbon border tax, the plastic tax). If progress is not made on these, countries' contributions to the Union budget will have to be increased.

There is little doubt that Next Generation EU is a turning point: for the first time, the Union is making a joint effort to revive growth based on the idea of temporary mutualisation of debt. What makes the agreement even more significant is Germany' position, which, from the outset, put all its weight behind the Commission's initiative. Nevertheless, it is certainly not a Hamiltonian moment, a founding act for a federal Europe. First, the RRF is temporary and does not take on existing debts; moreover, the "own resources" are currently only a wish list: Except for the plastic tax, there is a lack of agreement among member states on the taxation of multinationals, the Tobin tax and the carbon border tax.[1] In addition, investment programmes will remain national; it would have been desirable for this joint indebtedness effort to be put at the service of a vast European investment programme (Creel et al., 2020), but to date the EU does not have a spending capacity comparable to that of a federal state. In this sense, it is welcome news that the Commission has set strict guidelines for financing national plans, aimed at ensuring the overall coherence of national strategies and greater effectiveness in the supply of global public goods. Finally, the price to pay to get "frugal" countries on board has been a drastic reduction in the funding for European public goods such as education, the Invest Europe programme and health. For example, the proposal for a Health Union (the EU4Health programme) had been virtually defunded by the Council; only the commendable work by the European Parliament restored funding to €5 billion, about half of the (already modest) original proposal by the Commission. In a sort of institutional blur, while the RRF is geared towards adapting the European Union to the challenges of the post-pandemic world, the opportunity to direct the EU's ordinary instruments towards the same objective has been missed.

Nevertheless, if it is true that the RRF is a long way from being a federalist leap (for which, moreover, the political conditions are not met today), it is a game changer, and its impact on the EU landscape in the coming year might go well beyond the significant resources allotted to financing the recovery. What might remain, in fact, is the radical change of heart of the German government that follows the unprecedented fiscal effort of the spring 2020.

## The end of German virtue?

Following the global financial crisis, the cursor between the state and the market has moved towards the centre, and many economists today have no problem recognising a role for monetary and fiscal policies in smoothing the cycle and supporting growth. Moreover, the debate on multipliers and public investment has cleared the way for the idea that the sustainability of fiscal policy depends on its effect on growth as much as on public finances – a concept so trivial as to be oft forgotten during the eurozone crisis.

The first 20 years of the single currency and the sovereign debt crisis have shown that markets cannot do everything on their own. Yet, macroeconomic policies in the Economic and Monetary Union (EMU) have been inspired by the principle of risk reduction for a decade: The best way to avoid further crises and economic divergence would be to implement fiscal discipline and reforms in each country, following the idea that a chain is only as strong as its weakest link: The joint management of macroeconomic shocks would be unneces-

1 Remember that Alexander Hamilton in 1790 transferred the existing debt contracted by the states during the war of independence to the federal budget, financing its service with customs tariffs and an excise duty on whisky and other spirits, the first US federal tax.

sary if each country became resilient on its own. This is why, while fiscal consolidation in peripheral countries squeezed domestic demand and thus trade deficits, the core surpluses persisted and sometimes increased. The eurozone, entangled in a long deflationary spell, was gradually "Germanised": the current account surplus, close to zero until 2007, increased to a maximum of 3.6% of GDP in 2016.

The COVID-19 pandemic reshuffled the cards. International organisations are now warning against premature consolidations. OECD chief economist Laurence Boone recently noted that the mistake during the global financial crisis was not the lack of stimulus in 2009, but the rapid return to austerity from 2010 onwards. The IMF fiscal affairs department director Vitor Gaspar did not go as far as Boone and call the austerity of the 2010s a mistake. Nevertheless, presenting the IMF (2021) *Fiscal Monitor* last January, Gaspar insisted that today's low interest rates change the picture: while public debt in advanced economies doubled from 60% to 120% of GDP over the last 30 years, interest payments have halved from 4% of GDP to 2%, with obvious consequences for sustainability. Similarly, the European Commission noted that in the coming years, despite persistent sluggish growth, even more modest interest rate levels would lead to a downward trend in the ratio of public debt to GDP (Giles, 2021; IMF, 2021; European Commission, 2021).

The new intellectual environment is not the only reason to hope that the 2010 austerity drive will not return. The German change of attitude concerning fiscal policy and the mutualisation of efforts to fight the pandemic is driven by self-interest, and as such might be structural. Firstly, the international framework is different. During the Greek crisis, core EMU countries had looked with detachment at the convulsions of peripheral EMU countries. The collapse in their demand had been easily compensated by redirecting exports towards East Asia and the US. Thus, as long as financial stability was preserved, the health of the European market was of little interest to exporters in the core. Today things are different; there is no reason to think that the new US administration will act less aggressively than the previous one on trade, and world markets will not continue to absorb the surpluses of eurozone exporting countries. In the future, therefore, the European market, its prosperity, and its stable and balanced growth, will be in the best interest of Germany.

Besides the changed international environment, at home Germany is starting to feel the many years of "frugality". Between 2000 and 2019, both corporate and public investment in Germany were well below the eurozone average (OECD, 2020).[2] The resulting lack of capital creates bottlenecks and in the coming years will inevitably limit Germany's growth. In particular, firms' low knowledge-based investment and sluggish adoption of advanced ICTs hold back innovation and productivity growth. Population aging will do the rest, reducing the labour force and leading to a further squeeze on investment and labour productivity, eventually reducing potential growth. Last, but not least, the increase in inequality in recent years has disarticulated the social market economy model and helped to squeeze consumption and domestic demand.[3]

To sum up, today many contradictions of the "frugal" model come to the fore and the reforms of recent years have distorted the ordoliberal model, leaving behind a trail of precariousness and inequality. Themes such as trade surpluses, fiscal policy and wage policies, absent only a few years ago, are gaining momentum in the German policy discussions, which benefit from the emergence of a new generation of economists open to the international debate. The German daily *Süddeutsche Zeitung*'s 2015 survey of more than a thousand German economists showed that the intellectual landscape was changing rapidly (Fricke, 2015).[4] In the five years since the previous survey, for example, the proportion of economists who believed that fiscal policy plays a role in smoothing the cycle had risen from 18% to 36%. In addition, about two-thirds of respondents favoured the central bank role as a lender of last resort in the event of a financial crisis; this was right in 2015, as conflicts between Bundesbank President Jens Weidmann and ECB President Mario Draghi became public over the newly launched quantitative easing programme. The consensus is likely to have further evolved since then, with the COVID-19 crisis accelerating the movement. We saw that Germany was the European country that most decisively engaged in fiscal support of the economy, abandoning the balanced budget dogma (Hall, 2021). What is more, it was the German Ministry of Finance, a former temple of fiscal orthodoxy, which conceived the Merkel-Macron document in May 2020 recommending joint debt issuance, eventually leading to the Next Generation EU proposal.

The coming months will be key to understanding Germany's position in the European debate. Angela Merkel will not run in the 26 September general election. Many commentators believe that the most likely outcome is a

2 Dullien et al. (2020) estimate the need for public investment at €450 billion over the next ten years.
3 For more information, see the World Inequality Database, https://wid.world/country/germany/.
4 On the new generation of Keynesian economists who have gained influence in economic policy circles in Berlin in recent years, see Chazan (2020).

coalition between the CDU and the Green Party, which is likely to enjoy electoral success (Besch and Odendahl, 2021). In that case, investment and income distribution will be high on the agenda, even if the new CDU leader Armin Laschet will have to handle the rigorist wing of the party, which in the past has often resisted Merkel's choices (from the minimum wage to the green light to Next Generation EU).

## Towards a new role for Germany in eurozone reform?

The eurozone institutions have to be adapted to the newly found centrality of macroeconomic policy in ensuring macroeconomic stability and convergence. The coronavirus crisis proves that only real mutual insurance mechanisms, the features of a federal state, could guarantee stability and growth by operating together with (and sometimes compensating for) market adjustments. While it is obvious that today there is no leeway for a federal project, the existence of an ideal solution, however utopian, may be a useful benchmark. A German change of stance could allow progress in several reform areas that would have been unthinkable just one year ago.

### From mistrust to solidarity: Financial assistance and automatic stabilisation in the EMU

Over time, the EU has introduced several instruments for financial assistance to member states. A Jacques Delors Centre brief (Guttenberg, 2020) has recently stirred widespread discussion starting from the observation that the success of the SURE mechanism and (hopefully) of the Next Generation EU indicate a change of perspective: While financial assistance so far was based on mistrust among EU countries, the COVID-19 crisis has created political demand for solidarity and stabilisation mechanisms. The brief proposes to repatriate the ESM (now a sovereign bank governed by an intergovernmental treaty between eurozone countries) within the EU perimeter, as proposed by the Commission in 2017, and consolidate it with the plethora of other existing assistance instruments: SURE, the RRF loans scheme, the banks' Single Resolution Fund and the balance of payments assistance facility. The idea is to have a unique facility capable of offering credit lines differentiated by purpose and conditions of access. The ESM itself (that is today politically toxic) could then evolve into a debt agency (Amato et al., 2020) to coordinate and – in the case of joint projects such as Next Generation EU – mutualise national debts. Furthermore, it could issue that safe asset that would allow better management of European debt.

The reorganisation of the financial assistance facilities should go hand in hand with the creation of a tool for the reabsorption of asymmetric shocks. One such automatic stabiliser could be a European unemployment benefit scheme, proposed by several authors and endorsed by the European Commission (Andor, 2016; Arnold, 2018; Beblavý and Lenaerts, 2017). This should be a contingent scheme that would intervene in the event of significant deviations in the unemployment rate from a country-specific reference value, *in addition* to national programmes. This is obviously of paramount importance, because it would leave the choice of the extent and duration of protection against unemployment to individual countries' social contracts. Attempts to simulate the stabilisation capacity of the different proposals show that it could be generally designed in such a way that it does not lead to permanent transfers and that, in case of large shocks, its stabilisation capacity would be significant. Being specifically designed to absorb asymmetric shocks, on the other hand, it would not help in case of global shocks; to support all countries at once, the mechanism should be endowed with the capacity to borrow. In the past, the German Finance Minister Olaf Scholz has backed the idea of a European unemployment mechanism. Nevertheless, what he had in mind was a lending facility (along the lines of the recent SURE mechanism), rather than a proper insurance mechanism (Reuters, 2018).

Together with the introduction of a central stabilisation mechanism, future reform should strengthen the ability of markets to stabilise asymmetric shocks by deepening the capital markets union and the banking union. The divide among member states on this topic is not as deep as on rules or on central fiscal capacity. The only controversial point is the common deposit insurance, necessary to complete the banking union and to break the doom loop between sovereigns and financial institutions. This would introduce a limited amount of risk sharing and as such it has been met with hostility in Germany. However, before the pandemic, Germany had cautiously opened a window of opportunity that may have expanded further today (Scholz, 2019).

### A new fiscal rule

Absent a (politically unrealistic) federal budget, common stabilisation tools will unlikely provide enough shock absorption capacity to the EMU. National fiscal policy will remain central. This is why the most important debate of the next few months will be the one on the reform of the SGP. It would be simplistic to say that European fiscal rules forced pro-cyclical fiscal consolidation in the post-2010 period. Austerity is rather the political result of a narrative that traced the debt crisis back to the fiscal profligacy of the EMU peripheral countries. However, the institutions for macroeconomic governance were consistent with the shift towards austerity and, as the Greek crisis shows, provided the appropriate means of exerting pressure to impose it on recalcitrant governments.

Interestingly, the suspension of the SGP in March 2020 came a few weeks after the opening by the Commission of a consultation process on fiscal rules, which in turn was based on a surprisingly severe assessment of the existing framework (European Commission, 2020).[5] The Commission addressed criticisms put forward by independent economists since the SGP inception: The current framework (a) is excessively complex, arbitrary and difficult to enforce; (b) allows for the curbing of deficits but not debt, which is the real measure of public finances' sustainability; (c) penalises public investment, which is generally easier to reduce than current expenditure; (d) and finally, the European Commission (2020) recognised for the first time that the current framework forced procyclical deflationary policies (particularly between 2010 and 2013). In short, the Commission acknowledged, albeit implicitly, that European rules have made fiscal policy a source of instability and not of stabilisation.

The consultation process resumed in early 2021; as the SGP will remain suspended at least until 2022, it is safe to bet that the existing rules will be replaced before they come back into force. It is important that the new fiscal framework reconciles the objective of public finances' sustainability with the newfound centrality of budgetary policies in the policymaker's toolbox. The old idea of a golden rule of public finance, excluding investment from the deficit calculation, is once again making its way into the debate. The Commission recently announced that it is working on a proposal linking the rule to green investment (Euractiv, 2021). However, the pandemic showed once more the inadequacy of a purely accounting approach, identifying public investment with physical capital; following this approach, a large part of health expenditure, for example, would be considered current expenditure. The challenge would be to define investments in functional terms, to include all expenditure that increases not only physical capital, but also social and intangible capital, which are equally essential for growth. A political process involving the Commission, the Council and the Parliament would be central to the functioning of this "augmented golden rule" (Saraceno, 2017). Germany so far has been hostile to the idea of a golden rule; nevertheless, the already mentioned infrastructure gap and the colossal investment needs of the ecological transition might push towards a change of position, especially if the Green Party is part of the ruling coalition.

5 The Commission embraced the main conclusions of the European Fiscal Board (2019).

## Conclusion

The COVID-19 crisis has revived the economic policy debate in Europe, wiping out timidity and hesitation. In just a few months, tools for a common management of the crisis have been created that could eventually evolve into a completely different organisation of European macroeconomic policies. Interdependence and the need for risk-sharing instruments are now highlighted in areas such as health, public investment, ecological transition and the management of asymmetric shocks. While it is too early to say where this will lead, recent German actions are a reason to hope that in the next few years, the country will be an engine for reform rather than a stubborn defender of the status quo.
